# MT5-MMP, just a new APP processing proteinase in Alzheimer’s disease?

**DOI:** 10.1186/s12974-016-0633-4

**Published:** 2016-06-28

**Authors:** Kévin Baranger, Michel Khrestchatisky, Santiago Rivera

**Affiliations:** Aix Marseille Univ, CNRS, NICN, Marseille, France

**Keywords:** Matrix metalloproteinases, Neurodegenerative disease, Trafficking, Neuroinflammation, IL-1β, Amyloidogenesis, Amyloid precursor protein

## Abstract

We have recently identified in a transgenic mouse model of Alzheimer’s disease (AD) membrane-type 5-MMP (MT5-MMP) as a new player in Alzheimer’s pathogenesis, which displays pro-amyloidogenic features and proteolytic processing of amyloid precursor protein (APP). Another group has reported that MT5-MMP processing of APP may release a novel neurotoxic APP fragment. Although MT5-MMP-mediated APP processing appears to be a key pathogenic step, we hypothesize that MT5-MMP may also contribute to AD pathogenesis through complementary mechanisms that involve the activation of pro-inflammatory pathways and/or APP trafficking.

## Background

MT5-MMP belongs to the multigenic family of Zn^2+^ MMPs, which have been extensively associated with different physiological and pathological settings [1, 2]. MT5-MMP was first isolated from mouse brain tissue and from glioblastoma by two laboratories in 1999 [3, 4]. MT5-MMP is predominantly expressed in the nervous system [5], mainly in neurons, and to a lesser extent also in astrocytes and microglial and endothelial cells [6–8]. MT5-MMP is a 645 amino acid transmembrane glycosylated proteinase that is intracellularly activated by the Ca^2+^-dependent proprotein convertase furin. The latter can also cleave MT5-MMP above its transmembrane domain before it reaches the plasma membrane, thus leading to the release of a truncated active soluble form of MT5-MMP [9]. MT5-MMP harbors nuclear localization sequences that may tag the proteinase for import into the nucleus [10]. The proteolytic activity of MT5-MMP is mainly under the control of the endogenous tissue inhibitor of MMP-2 (TIMP-2). Alternatively, adaptor proteins such as Mint-3 regulate MT5-MMP activity by controling its recycling from the trans-golgi network to the cell membrane and its localization in cells [11]. Moreover, MT5-MMP can be targeted to synapses through interaction with proteins containing PDZ domains such as the AMPA receptor binding protein (ABP) and the glutamate receptor interacting protein (GRIP) [12]. Overall, MT5-MMP appears as a functionally versatile molecule by virtue of its multiple interactions and localizations in the intracellular and pericellular compartments. An open question is whether MT5-MMP may in turn influence the subcellular localization and activity of its interacting proteins, and should be considered as a “moonlighting protein.” One recent example among MMPs concerns MMP-12, which besides its well-known proteolytic activity has also transcription factor properties [13].

**Fig. 1:**
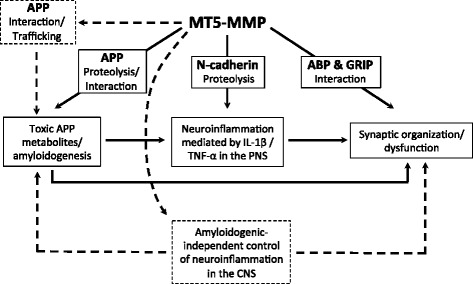
Scheme summarizing some known and some hypothetical functions of MT5-MMP in nervous system pathophysiology. *Solid lines/frames* represent biological actions and functional interactions reported in the literature. *Dotted lines/insets* represent the alternative/complementary hypotheses we propose herein. APP: amyloid precursor protein; ABP: AMPA receptor binding protein; CNS: central nervous system; GRIP: glutamate receptor interacting protein; IL-1β: interleukin-1 beta; PNS: peripheral nervous system; TNF-α: tumor necrosis factor-alpha

We are just starting to understand the functional diversity of MT5-MMP in pathology. Early studies showed the ability of MT5-MMP to proteolytically activate MMP-2 [3] and to process KiSS-1 protein and KISS-1-derived decapeptide metastin [14], which overall promotes cancer progression. MT5-MMP has been also reported to stimulate neuropathic pain by promoting aberrant axonal sprouting in the spinal cord after sciatic nerve injury in mice [15]. Moreover, it has been suggested that MT5-MMP is necessary for the inflammatory response to interleukin-1 beta (IL-1β) and tumor necrosis factor-alpha (TNF-α) in the peripheral nervous system [16]. It is noteworthy that no overt developmental abnormalities have been detected in MT5-MMP^−/−^ mice, in contrast with the clearly marked phenotypes they display in pathological conditions [15–17], and also in contrast with other members of the MT-MMP family such as MT1-MMP. This could imply that modulating MT5-MMP activity in pathology may have limited impact on physiology and highlights the therapeutic potential of targeting MT5-MMP.

## MT5-MMP in Alzheimer’s disease

MT5-MMP was first related to AD in a study showing the expression of the proteinase in dystrophic neurites around senile plaques in post-mortem AD brains [18]. The ability of MT5-MMP to process APP into different truncated APP fragments was later demonstrated [19]. More recently, two teams, including ours, reported the possible implication of MT5-MMP in AD by means of its functional interaction with APP [17, 20]. Willem and collaborators showed that MT5-MMP cleavage of APP upstream of the beta-site amyloid precursor protein cleaving enzyme 1 (BACE-1), combined with cleavage by the α-secretase ADAM-10, generates a so-called Aη-α fragment, which inhibits long-term potentiation (LTP) in vitro [20]. In parallel, we provided in vivo evidence that MT5-MMP deficiency is associated with a robust and durable amelioration of the pathological outcome in the 5xFAD transgenic mouse model of AD. Compared to 5xFAD mice, bigenic 5xFAD/MT5-MMP^−/−^ exhibited strong decreases of amyloid beta peptide (Aβ) load, gliosis and IL-1β levels, and a better preservation of the neuronal network. Moreover, cognitive and LTP dysfunctions were prevented in bigenic mice [17]. In the same study, we demonstrated that MT5-MMP interacts with APP and stimulates the formation of Aβ and the toxic C99 APP fragment. Together, these results unveil the contribution of MT5-MMP to AD pathogenesis and strongly suggest its involvement at least in APP processing. However, two complementary non-exclusive mechanistic hypotheses may be put forward on the pathogenic effects of MT5-MMP in AD:

## Hypotheses

1. The pro-amyloidogenic features of MT5-MMP could result from interactions with APP, which would stimulate its trafficking into endosomes, not necessarily in a proteolytic-dependent manner. These organelles are considered to be the main loci of Aβ production [21]. Testing this hypothesis would require to modulate the activity/presence of MT5-MMP by gain/loss of function in a cellular system and determine by imaging and biochemical approaches how this might change the distribution of APP and/or Aβ species in the endosomal/lysosomal compartments.
2. The pathogenic role of MT5-MMP in AD could also result from its contribution to the chronic neuroinflammatory process that aggravates AD pathogenesis. Reduced glial reactivity and IL-1β levels found in our bigenic 5xFAD/MT5-MMP^−/−^ mice [17] could simply result from MT5-MMP deficiency and consequent reduced Aβ load, but we cannot exclude that MT5-MMP inhibition could actually reduce neuroinflammation in an amyloid-independent manner. This idea is consistent with the two following findings: (a) intraplantar injections of IL-1β or TNF-α in MT5-MMP^−/−^ mice do not induce the pro-inflammatory response commonly observed in wild-type mice [16]. The underlying mechanism involves a deficient processing of N-cadherin, which is a substrate of MT5-MMP, and this appears to alter the communication between mast cells and peripheral sensory neurons. (b) The combined proteolytic action of MT5-MMP and α-secretase on APP can release under certain conditions a N-terminally elongated Aβ form, the neurotoxic Aη-α peptide [20]. Aη-α generation could have a neuroinflammatory effect associated with its neurotoxicity, but also a consubstantial reduction of the pool of soluble APP-α (sAPPα) generated by the solely action of α-secretase, and reported to be neuroprotective [22]. Whether MT5-MMP exerts pro-inflammatory activity in the brain could be tested by challenging neural cell cultures deficient for MT5-MMP with pro-inflammatory agents and determine whether well-known responsive genes are transcriptionally activated. Alternatively, the same experimental readout could be used after icv administration of the cytokines of interest in MT5-MMP^−/−^ brains. This idea could be further tested in young bigenic 5xFAD/MT5-MMP^−/−^ mice before Aβ accumulation.

## Implications of the hypotheses

The confirmation of the first hypothesis will uncover MT5-MMP as a multifunctional enzyme with proteolytic and non-proteolytic features, which could both ultimately stimulate APP metabolism and amyloidogenesis. The confirmation of the second hypothesis would add MT5-MMP to the list of MMPs that promote inflammation in the central nervous system and most interestingly would indirectly support the idea that neuroinflammation is a triggering event of the pathogenic process in AD. Confirming both hypotheses should place MT5-MMP at the crossroads of amyloidogenesis and neuroinflammation, thus providing ground for a better comprehension of AD pathophysiology (see Fig. 1). In turn, such confirmation would pave the way for the development of innovative therapeutic strategies based on the modulation of MT5-MMP, which should take into account the diversity of its biological actions.

## Abbreviations

5xFAD, transgenic mice expressing 5 human familial Alzheimer’s disease mutations; Aβ, amyloid beta peptide; ABP, AMPA receptor binding protein; ADAM-10, a disintegrin and metalloproteinase-10; APP, amyloid precursor protein; BACE-1, beta-site amyloid precursor protein cleaving enzyme 1; GRIP, glutamate receptor interacting protein; IL-1β: interleukin-1 beta; LTP, long-term potentiation; MMPs, matrix metalloproteinases; MT5-MMP, membrane-type 5-MMP; TIMP-2, tissue inhibitor of MMPs-2; TNF-α, tumor necrosis factor-alpha
